# Brain Sciences Special Issue: Neuroprotection against Ischemic Brain Injury

**DOI:** 10.3390/brainsci3031415

**Published:** 2013-09-24

**Authors:** Bruno P. Meloni

**Affiliations:** 1 Centre for Neuromuscular and Neurological Disorders, University of Western Australia, Crawley WA 6009, Australia; E-Mail: bruno.meloni@anri.uwa.edu.au; 2 Australian Neuromuscular Research Institute, Nedlands, WA 6009, Australia; 3 Department of Neurosurgery, Sir Charles Gairdner Hospital, Nedlands, WA 6009, Australia

It was my great pleasure to have acted as the guest editor for the Brain Sciences Special Issue on Neuroprotection against Ischemic Brain Injury. This Special Issue consists of a total of 18 articles covering a range of topics with the purpose of providing new knowledge and exploring novel interventions that one day may be used to better protect and repair the brain after ischemia. 

The topic of neuroprotection is especially important as in recent years there has been increasing concern regarding the failure of neuroprotective therapeutic modalities at clinical trial. Meanwhile, currently available therapies are not totally satisfactory, and are limited to clot removal (tPA thrombolysis, intra-arterial fibrinolysis, mechanical removal), aspirin and decompressive hemicraniectomy for ischemic stroke, and moderate hypothermia for cardiac arrest and perinatal hypoxia-ischemia. Despite these interventions there is still an urgent need to develop neuroprotective agents that can be given to a wider patient population and/or can boost the effectiveness of currently available treatments.

In this Special Issue about half of the articles focus on ischemic stroke, about a quarter on perinatal hypoxia-ischemia and the remainder on cerebral ischemia in general. Importantly, as all forms of brain ischemia have some overlap in terms of injury mechanisms and potential neuroprotective interventions the articles are likely to have a wide reader audience. Moreover, several articles are timely in terms of providing up-to-date information on topics relatively new to the field of neuroprotection namely stem cells, non-coding RNAs and mathematical modelling. Other articles review topics such as injury mechanisms and endovascular interventions, and involvement of mitochondria and NADPH oxidase in ischemic brain injury. The remainder of articles mainly examine specific neuroprotective molecules including peptides or proteins (substance P, ghrelin, nerve growth factor, prostacyclin IP receptor), and a range of diverse drugs (gilbenclamide, taurine, citicoline, cyclosporine A). 

Taken together, the scope of the subject matter in this Special Issue will in all likelihood be of interest to both new and experienced investigators involved in stroke research, whether preclinical or clinical. On this note, I would like to thank all the authors who have contributed to this Brain Sciences Special Issue on Neuroprotection against Ischemic Brain Injury.

